# Evaluating the Utility of Clinical Scores APACHE, CURB, SOFA, and NEWS2 at Admission and 5-Days after Symptom Onset in Predicting Severe COVID-19 in Patients with Diabetes

**DOI:** 10.3390/jpm14080868

**Published:** 2024-08-16

**Authors:** Radu Ion, Jaya Shankar Sai Kumar Kimidi, Chaitanya Kalapala, Oktrian FNU, Varshika Ramakrishnan Chandrababu, Omprakash Reddy Desireddygari, Mirela Loredana Grigras, Ovidiu Rosca, Felix Bratosin, Flavius Cioca, Romulus Timar, Rodica Anamaria Negrean

**Affiliations:** 1 Department III Functional Sciences, Division of Public Health and Management, Victor Babes University of Medicine and Pharmacy, 300041 Timisoara, Romania; radu.ion@umft.ro; 2 Katuri Medical College and Hospital, Katuri Health City 522019, India; jayakimidi@gmail.com; 3 Katuri Medical College, Dr YSR University of Health Sciences, Vijayawada 520008, India; kalapalachaitanya08827@gmail.com; 4 Faculty of Medicine, University of Indonesia, Jakarta 10430, Indonesia; oktrian@alumni.ui.ac.id; 5 Faculty of Medicine, Royal Stoke University Hospital, Stoke-on-Trent ST4 6QG, UK; varshika.chandrababu@uhnm.nhs.uk (V.R.C.); omprakash-reddy.desireddygari@uhnm.nhs.uk (O.R.D.); 6 Methodological and Infectious Diseases Research Center, Department of Infectious Diseases, “Victor Babes” University of Medicine and Pharmacy, Eftimie Murgu Square 2, 300041 Timisoara, Romania; grigoras.mirela@umft.ro (M.L.G.); ovidiu.rosca@umft.ro (O.R.); felix.bratosin@umft.ro (F.B.); 7 Doctoral School, “Victor Babes” University of Medicine and Pharmacy, Eftimie Murgu Square 2, 300041 Timisoara, Romania; 8 Discipline of Medical Statistics and Bioinformatics, “Victor Babes” University of Medicine and Pharmacy, Eftimie Murgu Square 2, 300041 Timisoara, Romania; 9 Department of Internal Medicine II, Division of Diabetes, Nutrition and Metabolic Diseases, “Victor Babes” University of Medicine and Pharmacy, Eftimie Murgu Square 2, 300041 Timisoara, Romania; timar.romulus@umft.ro; 10 Department of Preclinical Disciplines, Faculty of Medicine and Pharmacy, University of Oradea, 410073 Oradea, Romania; rodicanegrean@uoradea.ro

**Keywords:** diabetes mellitus, COVID-19, SARS-CoV-2

## Abstract

The elevated risk of severe COVID-19 outcomes in patients with diabetes underscores the need for effective predictive tools. This study aimed to assess the predictive accuracy of APACHE II, CURB-65, SOFA, and NEWS2 scores at critical time points in diabetic patients diagnosed with COVID-19, aiming to guide early and potentially life-saving interventions. In a prospective cohort study conducted from January 2021 to December 2023, adult patients with type 1 or type 2 diabetes and confirmed SARS-CoV-2 infection were evaluated. Clinical scores were calculated at admission and five days post-symptom onset, with data analyzed using receiver operating characteristic (ROC) curves and logistic regression to determine areas under the curve (AUC) and hazard ratios (HR) for severe outcomes. Among the 141 diabetic patients studied, ROC analysis revealed high AUC values for SOFA (0.771 at admission, 0.873 at day five) and NEWS2 (0.892 at admission, 0.729 at day five), indicating strong predictive accuracy for these scores. The APACHE II score’s AUC improved from 0.698 at admission to 0.806 on day five, reflecting worsening patient conditions. Regression analysis showed significant HRs associated with exceeding threshold scores: The SOFA score HR at day five was 3.07 (95% CI: 2.29–4.12, *p* < 0.001), indicating a threefold risk of severe outcomes. Similarly, the APACHE II score showed an HR of 2.96 (95% CI: 2.21–3.96, *p* < 0.001) at day five, highlighting its utility in predicting severe disease progression. The SOFA and NEWS2 scores demonstrated excellent early predictive accuracy for severe COVID-19 outcomes in diabetic patients, with significant AUC and HR findings. Continuous score monitoring, especially of APACHE II and SOFA, is crucial for managing and potentially mitigating severe complications in this vulnerable population. These tools can effectively assist in the timely escalation of care, thus potentially reducing morbidity and mortality among diabetic patients during the COVID-19 pandemic.

## 1. Introduction

The ongoing COVID-19 pandemic has posed significant challenges to healthcare systems globally, with certain populations, including patients with diabetes, experiencing markedly severe outcomes [1,2,3]. As of 2024, the intersection of COVID-19 with chronic diseases such as diabetes continues to draw considerable attention due to the elevated risk and worse prognosis in these patients. Studies have consistently shown that patients with diabetes are more likely to experience severe infections, require hospitalization, and face higher mortality rates compared to non-diabetic individuals [4,5,6].

In response to the critical need for early identification of patients at high risk of severe COVID-19, various clinical scores like APACHE II, CURB-65, SOFA, and NEWS2 have been adopted [7,8,9]. These scoring systems, originally designed to assess the severity of illness and predict outcomes in various clinical conditions, have been repurposed to address the unique challenges posed by COVID-19 [10,11,12,13]. The utility of these scores in the general population has been well documented; however, their effectiveness specifically in patients with diabetes, who are at an increased risk, remains under-explored.

Amidst the pandemic, the global prevalence of diabetes has been a contributing factor to the burden on healthcare systems [14]. Recent statistics indicate that over 10% of the global adult population is living with diabetes, with a significant number experiencing poor outcomes when infected with SARS-CoV-2 [15,16]. This highlights the need for robust predictive tools that can guide clinical decision-making and improve patient management, considering that diabetes also accounts for up to 10% of severe COVID-19 cases [17].

Given the dynamic nature of COVID-19, where patient conditions can deteriorate rapidly, the application of these clinical scores at different stages of the disease—specifically at admission and five days post-symptom onset—could provide critical insights. Current research indicates varying degrees of predictive accuracy for these scores, with some showing higher sensitivity and specificity in different patient subsets [18,19]. In diabetic patients, the absence of effective predictive tools often leads to suboptimal clinical decisions, exacerbating patient outcomes and increasing healthcare costs. For instance, without precise predictive models, clinicians may struggle to identify diabetic patients at high risk for severe complications like hypoglycemic events or cardiovascular issues, leading to either overtreatment or delayed intervention.

Therefore, this study aims to evaluate the predictive utility of APACHE II, CURB-65, SOFA, and NEWS2 scores at two critical time points in patients with diabetes diagnosed with COVID-19. By doing so, it seeks to determine which of these scores can most accurately predict severe outcomes and thus guide early and potentially life-saving interventions. The overarching objective is to refine these predictive tools to better serve high-risk populations, ultimately aiming to reduce the morbidity and mortality associated with COVID-19 in patients with diabetes.

## 2. Materials and Methods

### 2.1. Study Design and Ethics

This prospective cohort study evaluates the predictive accuracy of clinical scores for APACHE II, CURB-65, SOFA, and NEWS2 at admission and five days post-symptom onset in predicting severe COVID-19 outcomes in patients with diabetes. This study was conducted at the same hospital and included patients admitted from January 2021 to December 2023. Comprehensive data collection was performed using both electronic health records and manual chart reviews. Ethical approval was obtained from the institutional review board, adhering to the ethical standards of the Declaration of Helsinki, EU Good Clinical Practice Directives (2005/28/EC), and the International Council for Harmonization of Technical Requirements for Pharmaceuticals for Human Use (ICH) guidelines. This study was approved on 28 February 2022, with the approval number 05. All patient data were anonymized before analysis to ensure confidentiality.

### 2.2. Inclusion and Exclusion Criteria

Participants included in this study were adults aged 18 years and older with a confirmed diagnosis of type 1 or type 2 diabetes according to the American Diabetes Association guidelines. They were required to have a laboratory-confirmed SARS-CoV-2 infection, respiratory-predominant COVID-19 clinical form, verified by RT-PCR testing. Only patients with available data necessary for calculating the specified prognostic scores were included. Exclusion criteria encompassed patients with gestational diabetes or other unspecified diabetes forms, individuals with elevated serum glucose levels without a definitive diabetes diagnosis, including cases where the type of diabetes has not been clearly documented or diagnosed, and patient records that lacked specific details distinguishing between type 1 and type 2 and those with incomplete medical records lacking essential data like COVID-19 treatment details, outcome data, or HbA1c levels. Participants who did not consent to the use of their medical records for research purposes were also excluded.

This study used the World Health Organization (WHO) criteria for classifying COVID-19 severity, including mild cases, defined as individuals with uncomplicated symptoms and no severe pneumonia (SpO_2_ ≥ 94% on room air); moderate cases, characterized by signs of pneumonia but no severe pneumonia; severe cases, identified by respiratory distress, a high respiratory rate (>30 breaths/min), or low oxygen saturation (SpO_2_ < 90% on room air); and critical cases, involving respiratory failure, septic shock, or multiple organ dysfunction.

### 2.3. Data Collection and Variables

For this study, data collection included demographic information (age and gender) and diabetes-specific details such as type of diabetes (type 1 or type 2), disease duration, and HbA1c levels. COVID-19 severity was categorized based on the World Health Organization’s criteria, which classify cases as mild, moderate, or severe/critical. This classification takes into account symptoms, oxygen requirements, and chest imaging findings. Data on hospitalization, including ICU admissions and the necessity for mechanical ventilation, were also recorded. The Charlson Comorbidity Index (CCI) was utilized to evaluate the burden of comorbid conditions that could affect the prognosis of COVID-19 [20]. Mortality was noted as an outcome measure. The clinical scores APACHE II, CURB-65, SOFA, and NEWS2 were calculated at admission and five days post-symptom onset to assess their predictive accuracy for severe COVID-19 outcomes in diabetic patients.

### 2.4. Statistical Analysis

Data management and statistical analysis were performed using SPSS Statistics version 25.0. Continuous variables were expressed as means ± standard deviation (SD), while categorical variables were presented as frequencies and percentages. Comparison of continuous variables across different groups was performed using the Mann-Whitney U test due to the non-normal distribution of clinical score data, while categorical variables were compared using the Chi-square test. To determine the predictive power of the clinical scores, receiver operating characteristic (ROC) curves were plotted, and areas under the curve (AUC) were calculated for each clinical score separately, along with sensitivity and specificity values. Multiple logistic regression analysis was used to determine the odds ratios for severe COVID-19 outcomes based on the scores at admission and on day five. A *p*-value of less than 0.05 was considered statistically significant.

## 3. Results

In our study, we observed a total of 141 patients with diabetes and 316 without diabetes, who served as control. The average Body Mass Index (BMI) was notably higher in patients with diabetes (29.7 ± 4.7) compared to those without (27.4 ± 5.3), with a statistically significant difference (*p* < 0.001). Furthermore, a higher proportion of diabetic patients had a Charlson Comorbidity Index (CCI) greater than 2 (48.23% vs. 30.70%, *p* < 0.001), indicating a more complex health profile. Intensive Care Unit (ICU) admissions and the use of supplemental oxygen were also significantly more frequent among patients with diabetes (13.48% vs. 5.06%, *p* = 0.003 and 27.66% vs. 13.29%, *p* < 0.001, respectively). The analysis of outcomes related to COVID-19 severity showed no significant difference in the overall severity distribution between the two groups (*p* = 0.109). However, there was a higher incidence of mechanical ventilation usage in patients with diabetes (7.80% vs. 2.22%, *p* = 0.010) and a non-significant trend towards higher mortality (4.96% vs. 1.58%, *p* = 0.076), as presented in Table 1.

Patients with diabetes exhibited lower oxygen saturation levels (92.34 ± 3.21 vs. 94.56 ± 2.62, *p* < 0.001), higher white blood cell count (9.38 ± 2.82 × 10^9^/L vs. 7.21 ± 3.14 × 10^9^/L, *p* < 0.001), and elevated temperature (38.32 ± 0.72 °C vs. 37.98 ± 0.52 °C, *p* < 0.001) compared to those without diabetes. Additionally, significant differences were noted in heart rate, Glasgow coma scale, bilirubin levels, creatinine, PaO_2_/FiO_2_ ratio, platelet count, respiratory rate, systolic blood pressure, and blood urea nitrogen (BUN), all indicating more severe physiological impairment in patients with diabetes. Further analysis revealed that the clinical scores commonly used to assess the severity of illness—APACHE II, CURB-65, SOFA, and NEWS2—were significantly higher in the diabetic group compared to non-diabetics (APACHE II: 18.47 ± 6.32 vs. 15.13 ± 4.98, CURB-65: 2.14 ± 0.83 vs. 1.56 ± 0.69, SOFA: 4.03 ± 2.09 vs. 2.04 ± 1.22, NEWS2: 5.27 ± 1.98 vs. 3.29 ± 1.13, all *p* < 0.001), as presented in Table 2.

Five days post-symptom onset, our study found that patients with diabetes continued to exhibit worse physiological and clinical scores compared to those without diabetes, highlighting the prolonged impact of COVID-19 in this subgroup. Oxygen saturation levels remained significantly lower in patients with diabetes (90.78 ± 4.32) compared to those without (93.64 ± 3.58), with a *p*-value less than 0.001. Similarly, markers such as white blood cell count (11.22 ± 3.76 × 10^9^/L vs. 8.34 ± 2.99 × 10^9^/L), temperature (37.96 ± 0.82 °C vs. 37.12 ± 0.61 °C), and heart rate (92.38 ± 12.78 bpm vs. 84.56 ± 11.29 bpm) were significantly elevated in the diabetic group, all with p-values less than 0.001. Additionally, critical metrics such as the PaO_2_/FiO_2_ ratio, platelet count, and levels of bilirubin and creatinine were notably worse in diabetic patients, indicating severe ongoing physiological stress and organ dysfunction.

Furthermore, the clinical scores used to assess severity and predict outcomes—APACHE II, CURB-65, SOFA, and NEWS2—were also significantly higher in diabetic patients compared to their non-diabetic counterparts (all *p*-values < 0.001). Specifically, APACHE II scores increased to 19.52 ± 7.23 from 18.47 ± 6.32, and SOFA scores rose to 4.56 ± 2.31 from 4.03 ± 2.09, reflecting an escalation in the severity of illness in diabetic patients over the initial five days of symptoms. This worsening trend was also mirrored in CURB-65 and NEWS2 scores, underscoring the heightened risk and the predictive value of these scores in identifying patients who may require more intensive care or may face a higher risk of adverse outcomes (Table 3).

The analysis of best cutoff values for predicting severe COVID-19 outcomes in patients with diabetes revealed statistically significant sensitivity, specificity, and area under the curve (AUC) values for all evaluated clinical scores at both admission and 5 days after symptom onset. At admission, the SOFA score exhibited high diagnostic accuracy with an optimal cutoff of 3.43, achieving a sensitivity of 89.67% and a specificity of 90.43%, with an AUC of 0.771 (*p* < 0.001). Similarly, the NEWS2 score demonstrated a robust predictive value with a cutoff of 4.22, yielding a sensitivity of 82.67% and a specificity of 80.29%, reflected in a high AUC of 0.892 (*p* < 0.001). These results suggest that the SOFA and NEWS2 scores are particularly effective in early identification of severe disease among patients with diabetes.

Further assessment five days post-symptom onset showed an increase in the predictive performance of the clinical scores. The APACHE II score’s cutoff increased to 19.26, with improved sensitivity and specificity (90.31% and 91.57%, respectively), and an AUC of 0.806 (*p* < 0.001). The SOFA score remained a strong predictor with a new cutoff of 3.72, exhibiting even higher sensitivity and specificity of 92.47% and 93.12%, respectively, and an AUC of 0.873 (*p* < 0.001), as presented in Table 4.

The regression analysis conducted to assess the risk of developing severe COVID-19 in patients with diabetes, based on clinical scores exceeding their respective best cutoff values, demonstrated significant associations across all scores both at admission and 5 days post-symptom onset. At admission, the SOFA score was associated with the highest hazard ratio (HR) of 2.82 (95% CI: 2.10–3.78, *p* < 0.001), indicating a nearly threefold increased risk of severe disease progression in patients scoring above the threshold compared to those below. Similarly, the APACHE II score at admission also showed a significant increased risk with an HR of 2.47 (95% CI: 1.83–3.32, *p* < 0.001). 

**Figure 1:**
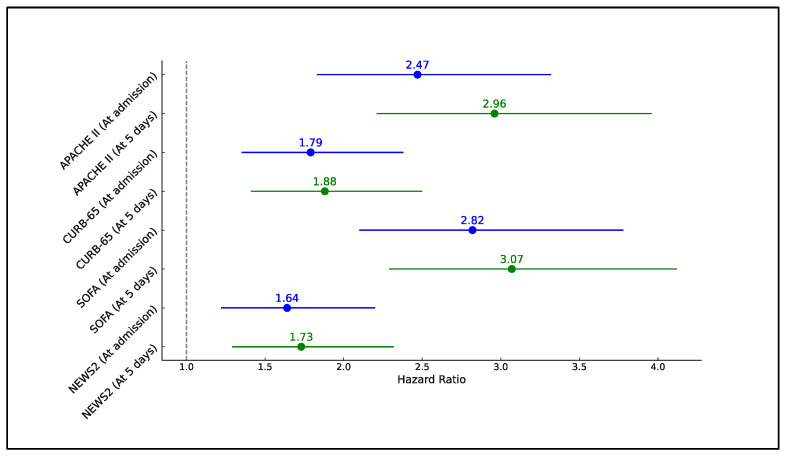
Forest plot analysis of risk of COVID-19 development in patients with diabetes.

Five days after symptom onset, the predictive power of the clinical scores increased further, suggesting that continuous monitoring of these scores can provide crucial insights into patient deterioration. The SOFA score continued to show the highest HR at 3.07 (95% CI: 2.29–4.12, *p* < 0.001), followed by APACHE II with an HR of 2.96 (95% CI: 2.21–3.96, *p* < 0.001). The results for CURB-65 and NEWS2 also indicated a substantial risk of severe COVID-19 with HRs of 1.88 (95% CI: 1.41–2.50, *p* < 0.001) and 1.73 (95% CI: 1.29–2.32, *p* < 0.001), respectively (Table 5 and Figure 1).

## 4. Discussion

### 4.1. Analysis of Findings

This study provides significant insights into the predictive efficacy of clinical scoring systems like APACHE II, CURB-65, SOFA, and NEWS2 for severe COVID-19 outcomes in patients with diabetes. The results underscore a critical need for specialized monitoring and management strategies tailored to patients with diabetes who are at increased risk during the COVID-19 pandemic. Notably, the elevated clinical scores at admission highlight the initial severity of the infection in individuals with diabetes, underscoring their predisposition to more severe disease trajectories compared to non-diabetic individuals. The consistently higher values in physiological parameters such as BMI, oxygen saturation, and biomarkers of inflammation further delineate the heightened vulnerability of this group, thus emphasizing the utility of these scores in early risk stratification.

The evolution of clinical scores from admission to five days post-symptom onset illustrates an aggravation in clinical status for patients with diabetes, as evidenced by increased scores across all metrics. This trend was particularly pronounced for the SOFA score, which was associated with the highest hazard ratios both at admission and after five days, suggesting its superior predictive value for critical outcomes. The increase in SOFA and APACHE II scores over time also indicates progressive organ dysfunction, warranting aggressive and preemptive therapeutic interventions to mitigate the risk of severe outcomes, including ICU admissions and mechanical ventilation.

Moreover, the study’s findings on the best cutoff values of these scores at two distinct time points provide a practical framework for clinicians. These thresholds can be instrumental in the timely identification of patients with diabetes who are likely to benefit from escalated care, thereby potentially reducing the morbidity and mortality associated with COVID-19 in this high-risk population. The high sensitivity and specificity of these scores, particularly the SOFA and NEWS2 scores, reinforce their reliability and essential roles in the clinical decision-making process, guiding the intensification of care where needed.

In the study by Mehryar et al. [21], the efficacy of the APACHE II scoring system was evaluated for predicting mortality in COVID-19 ICU patients, revealing a mean score of 10.12 ± 6.3 among 150 patients. Conditions such as cough, shortness of breath, and renal failure were linked to higher scores, though the score did not reliably predict mortality. Similarly, Plummer et al. [22] investigated the impact of diabetes on COVID-19 severity in Australian ICUs, finding that 43% of the patients had diabetes, which was associated with higher APACHE II scores, longer hospital stays, and greater glycemic variability, with an overall hospital mortality of 16%. Both studies underscore the challenges of using clinical scores to predict outcomes in critically ill COVID-19 patients, highlighting the need for nuanced management strategies, particularly for vulnerable populations with pre-existing conditions like diabetes.

The two studies by Asmarawati et al. [23] and Beigmohammadi et al. [24] delve into the utility of clinical scoring systems such as APACHE II and SOFA for predicting mortality in COVID-19 patients in ICU settings. Asmarawati et al. conducted a prospective cohort study with 53 patients, finding that on day 5, the qSOFA, SOFA, APACHE II, and NEWS-2 scores significantly differentiated between survivors and non-survivors. Notably, APACHE II exhibited the highest sensitivity (95.7%) and specificity (86.7%) for predicting mortality on day 5. Similarly, Beigmohammadi et al. assessed 204 patients and reported that mean SOFA and APACHE II scores were significantly higher in non-survivors than survivors, with the SOFA score showing a superior area under the curve (89.5%) compared to APACHE II (73%). Both studies confirm the relevance of these scoring systems in predicting outcomes, although Beigmohammadi et al. suggest the need for more refined scores that consider daily clinical changes to enhance predictive accuracy.

The studies conducted by Esmaeili Tarki et al. [25] and Yang et al. [26] both evaluate the prognostic utility of the Sequential Organ Failure Assessment (SOFA) score in predicting outcomes for COVID-19 patients in ICU settings, highlighting its potential in clinical assessments. Esmaeili Tarki et al. conducted a prospective cohort study involving 1057 patients, determining that the mean SOFA score during the first 96 h had a strong association with 28-day mortality (HR: 3.82), asserting its utility over other time-specific SOFA measurements. This finding is complemented by Yang et al.’s retrospective study of 117 patients, which found that the SOFA score not only differentiated between severe and mild COVID-19 cases but also possessed a high diagnostic accuracy for predicting severe outcomes (AUC = 0.908) and death risk (AUC = 0.995). Both studies underscore the SOFA score’s robustness as a predictive tool in the ICU, with Yang et al. further establishing its efficacy in a retrospective setting and Esmaeili Tarki et al. demonstrating its dynamic utility when measured over the first 96 h of ICU admission.

Belikina et al. [27] conducted a case-control study with 64 patients to examine the interaction between COVID-19 and diabetes mellitus, finding that patients with DM experienced more severe symptoms, including extensive lung damage, higher mortality risk as per the CURB-65 algorithm, and prolonged periods of hypoxia. They also reported elevated levels of inflammation markers and indicators of hypercoagulability, such as C-reactive protein and D-dimer. Similarly, Guo et al. [28] assessed the CURB-65 score’s predictive accuracy in a retrospective cohort of 74 patients, confirming its effectiveness in predicting in-hospital death with an area under the curve (AUC) of 0.81. Both studies highlight the importance of specific clinical scores and markers in managing COVID-19 patients, with Belikina et al. underscoring the compounded risk in patients with diabetes and Guo et al. demonstrating the CURB-65 score’s utility in triaging severe cases, thus providing crucial insights for better clinical decision-making during the pandemic.

Eldaboosy et al. [29] conducted a multicenter retrospective study on 1131 patients in Saudi Arabia to compare the effectiveness of the SIPF (shock index and hypoxemia), CURB-65, and APACHE II scores in predicting in-hospital mortality and ICU admission. They found that the SIPF score had a significantly higher predictive accuracy for both ICU admission (AUC 0.89) and mortality (AUC 0.90) compared to CURB-65 and APACHE II. In a similar manner, the study by Nikniaz et al. [30] analyzed the outcomes of 317 hospitalized patients with diabetes with COVID-19 in Iran, focusing on the impact of obesity. Their findings highlighted that obesity significantly increased the risk of death, ICU admission, and ventilator dependence among patients with diabetes, with obese patients being 2.72 times more likely to die than non-obese patients. Both studies underscore the importance of specific patient characteristics—like shock index and obesity—in refining the prognosis and treatment strategies for COVID-19, thereby enhancing clinical decision-making processes in pandemic responses.

The implications of our findings in light of previous research suggest that while our results align with established studies, further exploration of discrepancies could yield valuable insights. Variations in predictive accuracy of scores such as APACHE II, CURB-65, SOFA, and NEWS2 may stem from differences in study populations, methodologies, or clinical environments. This understanding prompts us to propose a practical framework for utilizing these clinical scores to effectively identify patients with diabetes at increased risk for severe complications from COVID-19. By establishing and applying specific cut-off values for each score, these findings can facilitate the early detection of at-risk individuals, thereby improving patient management and potentially reducing mortality rates during the pandemic.

### 4.2. Study Limitations and Future Perspectives

One of the main limitations of this study is the relatively small sample size of patients with diabetes, which may limit the generalizability of the findings to all patients with diabetes and COVID-19. Additionally, the study’s setting in a single hospital might introduce selection bias and limit the applicability of the results to other healthcare environments with different patient demographics and treatment protocols. The retrospective nature of part of the data collection could also result in information biases, particularly concerning the accuracy and completeness of the recorded data. Furthermore, this study does not account for potential confounders such as variations in treatment regimens over time or the introduction of new therapies during the study period, which might influence outcomes independently of the prognostic scores evaluated.

However, the critical analysis of this study also reveals areas needing further investigation, such as the impact of varying levels of glycemic control and other diabetes-related complications on the effectiveness of these scoring systems. Future research should aim to refine these predictive tools by incorporating diabetes-specific factors, which could enhance their accuracy. Additionally, longitudinal studies are necessary to track long-term outcomes and validate the predictive power of these scores beyond the acute phase of the infection, ultimately aiding in the development of more robust, diabetes-centric predictive models for infectious diseases like COVID-19. Future studies should also investigate targeted interventions on identified key variables to assess their direct impact on outcomes, enhancing our understanding of causal relationships and potential treatment efficacy. Lastly, to enhance predictive accuracy for severe outcomes in patients with diabetes, the creation of diabetes-specific scores is proposed. This initiative would aim to tailor predictive tools more closely to the needs of diabetic populations, thereby improving management strategies and potentially decreasing morbidity and mortality in this high-risk group.

## 5. Conclusions

This study concluded that among the various clinical scores assessed—APACHE II, CURB-65, SOFA, and NEWS2—the SOFA and NEWS2 scores, especially when assessed five days post-symptom onset, provided the most reliable predictions of severe outcomes in patients with diabetes diagnosed with COVID-19. Integrating these findings into clinical practice could involve updating treatment protocols to include periodic reassessment of these scores, thereby facilitating dynamic and responsive patient management strategies that can adapt to the evolving severity of the disease.

## Figures and Tables

**Table 1:** Demographic and Clinical Characteristics of Patients with and without Diabetes.

Variables	With Diabetes (*n* = 141)	Without Diabetes (*n* = 316)	*p*-Value
Age, years (mean ± SD)	58.7 ± 13.4	56.2 ± 14.1	0.076
Gender, men	82 (58.16%)	153 (48.42%)	0.068
BMI (mean ± SD)	29.7 ± 4.7	27.4 ± 5.3	<0.001
Smoking	28 (19.86%)	63 (19.94%)	0.986
Alcohol use	13 (9.22%)	36 (11.39%)	0.596
Diabetes type	-	-	-
T1DM	22 (15.61%)	-	-
T2DM	119 (84.39%)	-	-
Duration of diabetes, years (mean ± SD)	8.1 ± 6.6	-	-
HbA1C (mean ± SD)	8.4 ± 2.9	-	-
COVID-19 vaccinated	77 (54.61%)	159 (50.32%)	0.455
CCI > 2	68 (48.23%)	97 (30.70%)	<0.001
COVID-19 severity	-	-	0.109
Mild	63 (44.68%)	167 (52.85%)	-
Moderate	51 (36.17%)	84 (26.58%)	-
Severe	27 (19.15%)	65 (20.57%)	-
ICU admissions	19 (13.48%)	16 (5.06%)	0.003
Supplemental oxygen	39 (27.66%)	42 (13.29%)	<0.001
Mechanical ventilation	11 (7.80%)	7 (2.22%)	0.010
Mortality	7 (4.96%)	5 (1.58%)	0.076

SD—Standard Deviation; BMI—Body Mass Index; T1DM—Type 1 Diabetes Mellitus; T2DM—Type 2 Diabetes Mellitus; CCI—Charlson Comorbidity Index; ICU—Intensive Care Unit.

**Table 2:** Baseline Clinical Scores and Physiological Parameters at Admission.

Variables (Mean ± SD)	With Diabetes (*n* = 141)	Without Diabetes (*n* = 316)	*p*-Value
Oxygen saturation	92.34 ± 3.21	94.56 ± 2.62	<0.001
WBC (×10^9^/L)	9.38 ± 2.82	7.21 ± 3.14	<0.001
Temperature	38.32 ± 0.72	37.98 ± 0.52	<0.001
Heart rate	88.42 ± 11.24	81.37 ± 13.19	<0.001
Glasgow coma scale	14.22 ± 1.63	14.95 ± 0.42	<0.001
Bilirubin levels	1.34 ± 0.56	0.92 ± 0.31	<0.001
Creatinine (mg/dL)	1.64 ± 0.61	0.95 ± 0.42	<0.001
PaO_2_/FiO_2_ ratio	285.43 ± 65.23	320.47 ± 50.35	<0.001
Platelets	220.39 ± 50.67	250.28 ± 70.43	<0.001
Respiratory rate	22.15 ± 3.97	19.06 ± 3.09	<0.001
Systolic blood pressure	130.87 ± 15.32	125.48 ± 19.76	0.004
BUN	29.42 ± 10.38	18.65 ± 8.24	<0.001
Clinical scores			
APACHE II	18.47 ± 6.32	15.13 ± 4.98	<0.001
CURB-65	2.14 ± 0.83	1.56 ± 0.69	<0.001
SOFA	4.03 ± 2.09	2.04 ± 1.22	<0.001
NEWS2	5.27 ± 1.98	3.29 ± 1.13	<0.001

SD—Standard Deviation; WBC—White Blood Cell; BUN—Blood Urea Nitrogen; APACHE II—Acute Physiology and Chronic Health Evaluation II; CURB-65—Confusion, Urea, Respiratory rate, Blood pressure, Age ≥ 65; SOFA—Sequential Organ Failure Assessment; NEWS2—National Early Warning Score 2.

**Table 3:** Clinical Scores and Physiological Parameters at 5 Days Post-Symptom Onset.

Variables (Mean ± SD)	With Diabetes (*n* = 141)	Without Diabetes (*n* = 316)	*p*-Value
Oxygen saturation	90.78 ± 4.32	93.64 ± 3.58	<0.001
WBC (×10^9^/L)	11.22 ± 3.76	8.34 ± 2.99	<0.001
Temperature	37.96 ± 0.82	37.12 ± 0.61	<0.001
Heart rate	92.38 ± 12.78	84.56 ± 11.29	<0.001
Glasgow coma scale	13.87 ± 1.94	14.89 ± 0.83	<0.001
Bilirubin levels	1.52 ± 0.67	0.89 ± 0.36	<0.001
Creatinine (mg/dL)	1.82 ± 0.73	0.98 ± 0.47	<0.001
PaO_2_/FiO_2_ ratio	268.94 ± 73.48	315.72 ± 51.23	<0.001
Platelets	205.14 ± 55.23	244.38 ± 69.47	<0.001
Respiratory rate	24.21 ± 5.18	20.84 ± 4.21	<0.001
Systolic blood pressure	128.65 ± 16.42	122.94 ± 18.34	0.002
BUN	31.78 ± 11.25	20.39 ± 9.28	<0.001
Clinical scores			
APACHE II	19.52 ± 7.23	16.49 ± 6.17	<0.001
CURB-65	2.38 ± 0.92	1.62 ± 0.76	<0.001
SOFA	4.56 ± 2.31	2.38 ± 1.32	<0.001
NEWS2	5.92 ± 2.16	3.84 ± 1.47	<0.001

SD—Standard Deviation; WBC—White Blood Cell; BUN—Blood Urea Nitrogen; APACHE II—Acute Physiology and Chronic Health Evaluation II; CURB-65—Confusion, Urea, Respiratory rate, Blood pressure, Age ≥ 65; SOFA—Sequential Organ Failure Assessment; NEWS2—National Early Warning Score 2.

**Table 4:** Best Cutoff Values for Severe COVID-19 Prediction in Patients with Diabetes.

Parameters	Time Frame	Best Cutoff Value	Sensitivity	Specificity	AUC	*p*-Value
APACHE II	At admission	17.34	87.21%	84.62%	0.698	<0.001
CURB-65	At admission	2.18	81.14%	79.48%	0.883	<0.001
SOFA	At admission	3.43	89.67%	90.43%	0.771	<0.001
NEWS2	At admission	4.22	82.67%	80.29%	0.892	<0.001
APACHE II	At 5 days	19.26	90.31%	91.57%	0.806	<0.001
CURB-65	At 5 days	2.44	84.19%	83.24%	0.780	<0.001
SOFA	At 5 days	3.72	92.47%	93.12%	0.873	<0.001
NEWS2	At 5 days	5.16	85.83%	86.37%	0.729	<0.001

APACHE II—Acute Physiology and Chronic Health Evaluation II; CURB-65—Confusion, Urea, Respiratory rate, Blood pressure, Age ≥ 65; SOFA—Sequential Organ Failure Assessment; NEWS2—National Early Warning Score 2.

**Table 5:** Regression Analysis for Severe COVID-19 Development in Patients with Diabetes.

Factors above the Best Cutoff	Time Frame	Hazard Ratio	95% CI	*p*-Value
APACHE II	At admission	2.47	1.83–3.32	<0.001
CURB-65	At admission	1.79	1.35–2.38	<0.001
SOFA	At admission	2.82	2.10–3.78	<0.001
NEWS2	At admission	1.64	1.22–2.20	<0.001
APACHE II	At 5 days	2.96	2.21–3.96	<0.001
CURB-65	At 5 days	1.88	1.41–2.50	<0.001
SOFA	At 5 days	3.07	2.29–4.12	<0.001
NEWS2	At 5 days	1.73	1.29–2.32	<0.001

APACHE II—Acute Physiology and Chronic Health Evaluation II; CURB-65—Confusion, Urea, Respiratory rate, Blood pressure, Age ≥ 65; SOFA—Sequential Organ Failure Assessment; NEWS2—National Early Warning Score 2.

## Data Availability

The data presented in this study are available on request from the corresponding author.
